# Modifying Post-Surgical Immunity: Controlled Release of TLR7/8 Agonist for Immune Mediated Clearance of Glioblastoma

**DOI:** 10.21203/rs.3.rs-5024510/v1

**Published:** 2024-09-26

**Authors:** Kristy Ainslie

**Affiliations:** The University of North Carolina at Chapel Hill

## Abstract

Glioblastoma is an aggressive brain cancer with a dismal prognosis despite current therapeutic interventions. Tumor resection is standard-of-care for glioblastoma and has profound immunostimulatory effects. Resulting in a nadir in tumor burden, resection offers a unique opportunity to break local immune tolerance and mount an effective anti-tumor immune response. Here, we explore the effect of local and controlled release of TLR7/8 agonist from a polymer scaffold implanted at the time of tumor resection. We find that sustained release of TLR7/8 agonist leads to clearance of residual post-resection tumor, improved survival, and subsequent protection from tumor challenge in mice bearing orthotopic GL261 or CT2A gliomas. We show that scaffold therapy boosts resection-mediated disruption to the tumor microenvironment, leading to an early inflammatory innate immune response both in the brain and cervical lymph node. This is followed by an influx of activated NK cells in the brain and effector T cells in the lymph node and brain. In sum, sustained local TLR7/8 agonism within the context of tumor resection is a promising approach for glioblastoma.

## INTRODUCTION

Glioblastoma (GBM) is a highly invasive and aggressive brain tumor. Even with standard-of-care therapy including surgical resection, radiation, and chemotherapy, local tumor recurrence and mortality is almost 100%.(1–3) In an effort to combat local tumor recurrence, extensive research has evaluated drug delivery systems implanted directly in the post-surgical cavity to locally deliver chemotherapies.(4–6) A clinical example of this is Gliadel^®^, an FDA approved biodegradable polymeric wafer that delivers carmustine directly into the brain tissue of the tumor resection cavity.(7, 8) However, limitations with drug diffusion kinetics and a high rate of adverse events have restricted its efficacy.(9)

Immunotherapies are a promising alternative to cytotoxic chemotherapies, and their success against other malignancies has sparked exploration for GBM. Unfortunately, GBM immunotherapy faces a steep uphill battle.(10–16) GBM has one of the most immunosuppressive tumor microenvironments (TME), (17, 18) but also elicits a variety of systemic immune derangements, such as T cell sequestration in the bone marrow.(19) Additionally, GBM has a low tumor mutational burden which complicates the identification of neoantigens.(20) Moreover, single antigen approaches are often confounded by the heterogenous nature of GBM.(21–23)

One strategy for combatting the immunosuppressive TME has been to target microglia and macrophages which are major mediators of anti-inflammatory signaling in GBM.(24, 25) Microglia and macrophages express substantial levels of Toll-like receptors (TLRs), innate immune sensors which recognize pathogen-associated molecular patterns (PAMPs).(24) Activation of TLRs has been a useful therapeutic avenue for cancer therapy, capable of shifting immunosuppressive cells towards a pro-inflammatory phenotype as well as activating both innate and subsequently adaptive immune responses against tumors.(26) However, preclinical investigations into TLR agonists for GBM therapy have had varied success.(27, 28)

Surgical debulking of the tumor is a core component of GBM clinical treatment and results in significant immunological effects. Studies have shown that trauma caused by surgery and the resulting immediate inflammation can later exacerbate immunosuppression within the TME.(29, 30) However, in the immediate term, GBM surgery reduces myeloid-derived suppressor cells (MDSC) and promotes recruitment of effector T cells to the brain.(31, 32) This suggests that perioperative immunotherapy could both capitalize on the reduced tumor burden and disrupted TME, as well as potentially mitigate concerns of later surgical-induced tumorigenesis.(33) Indeed, neoadjuvant administration of checkpoint blockade has already demonstrated some promise in GBM.(34)

Immunotherapy delivered directly to the tumor site has proven safer and more effective than systemic administration.(35) By focusing delivery near the tumor, local immune tolerance can be disrupted, facilitating systemic antitumor immunity. One way this can be achieved is by loading an immunotherapeutic within a biodegradable polymer implant.(36–39) Through diffusion and polymer matrix degradation, the immunotherapeutic is released at a sustained rate creating a local inflammatory response and chemotactic gradient to recruit leukocytes to the brain. Additionally, the polymer matrix can shield the encapsulated immunotherapeutic and limit drug degradation *in situ*.(40, 41)

In this study, we formulated a small molecule TLR7/8 agonist, resiquimod, within a biodegradable acetalated dextran (Ace-DEX) scaffold which can offer controlled release of resiquimod from the polymeric nanofibrous matrix. We evaluated the differential pharmacokinetics of bolus versus scaffold administration of resiquimod placed in a sham resection cavity in the brain. In two murine models of tumor resection and recurrence, GL261 and CT2A, we compared scaffold therapy to a local bolus injection of resiquimod, both applied immediately post-tumor debulking. Surviving mice were challenged with the tumor to determine the formation of immune memory. Finally, we explored the local and systemic immune response in the immediate days following tumor resection and treatment with scaffold therapy.

## RESULTS

### Sustained delivery of resiquimod leads to tumor clearance and immunologic memory

**Figure 1:**
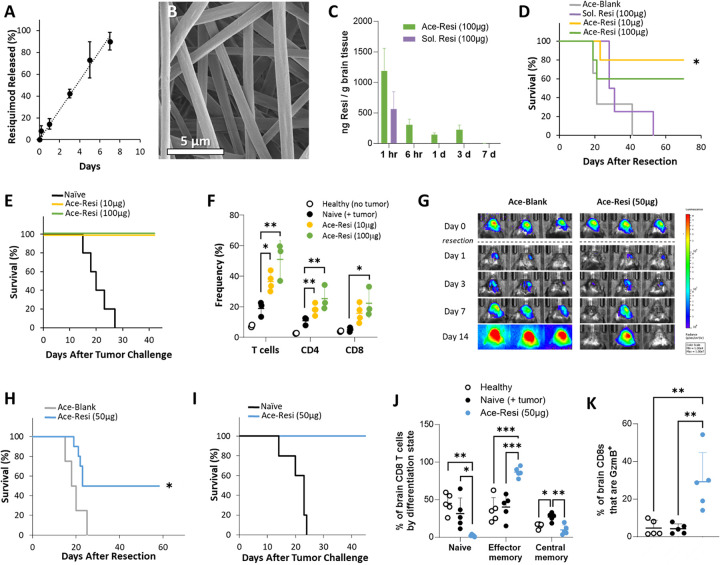
GL261 study and pharmacokinetics **(A)** Release of resiquimod from polymer scaffold in vitro. **(B)** Scanning electron micrograph of Ace-Resi scaffold. **(C)** Concentration of resiquimod in the brain over time for mice treated with 100 μg of resiquimod via Ace-Resi or bolus injection (Sol. Resi). **(D)** Survival of mice after tumor resection and treatment with Ace-Blank, soluble resiquimod (100 μg), Ace-Resi (10 μg), or Ace-Resi (100 μg). *p<0.05 by Log-Rank test with respect to Ace-Blank and Sol. Resi groups. **(E)** Survival of mice after first tumor challenge. **(F)** Frequency of T cells in the brain 1 week after second tumor challenge. *p<0.05, **p<0.01 one-way ANOVA with multiple comparisons Dunnett’s post-hoc test. **(G)** Representative bioluminescent images of mice after tumor resection. **(H)** Survival of mice after tumor resection and treatment with vehicle Ace-Blank or Ace-Resi (50 μg). *p<0.05 by Log-Rank test. **(I)** Survival of mice after first tumor. **(J)** Differentiation state of CD8^+^ T cells in the brain one week after second tumor challenge. **(K)** Percentage of granzyme B^+^ CD8^+^ T cells in the brain one week after second tumor challenge. *p<0.05, **p<0.01, ***p<0.001 by one-way ANOVA with multiple comparisons Dunnett’s post-hoc test.

To probe the benefit of sustained local TLR7/8 stimulation within the context of tumor resection, resiquimod was formulated within an Ace-DEX polymer scaffold for controlled release. Ace-DEX is a biodegradable polymer with adjustable degradation rates created by modifying hydroxyl groups of dextran with cyclic and acyclic acetals.(42–44) Resiquimod was incorporated within an Ace-DEX polymer scaffold through electrospinning. The resulting resiquimod loaded scaffold, ‘Ace-Resi’, was composed of randomly aligned microfibers and displayed zero-order release of resiquimod over 7 days *in vitro* (Fig. 1A, B). The scaffold was completely degraded by 14 days *in vitro* (**Supp** Fig. 1A).

The pharmacokinetics of Ace-Resi (100 μg) scaffolds compared to a bolus injection of 100 μg resiquimod in phosphate buffered saline (Sol. Resi) were evaluated in healthy mice (no intracranial tumor). Ace-Resi scaffolds were implanted or Sol. Resi injected within a sham resection cavity in the right hemisphere of the brain. This is important, as studies have shown that resection can greatly affect drug diffusion.(45, 46) Ace-Resi scaffolds resulted in a higher concentration of resiquimod in the whole brain compared to bolus injection throughout a seven-day period (Fig. 1C), increasing the area under the curve (AUC) in the brain by 19-fold (**Supp Table 1**). Plasma resiquimod concentration for both the scaffold and bolus injection was maximum at the 1-hour timepoint, tapering off quickly (**Supp** Fig. 1B). When comparing resiquimod concentrations in whole brain to plasma out to day 7, the concentration in brain was 8.0 to 18.9-fold higher than in plasma for Ace-Resi, which is evidence for prolonged local drug retention mediated by the scaffold (**Supp** Fig. 1C). In contrast, bolus injection only exhibited a 4.6-fold higher concentration of resiquimod in the brain at 1 hour, and by 6 hours it was roughly equivalent to plasma. Additionally, we evaluated the retention of resiquimod in Ace-Resi scaffolds removed from brains during this *in vivo* study to compare to *in vitro* conditions (**Supp** Fig. 1D) and found that resiquimod is released slightly faster *in vivo*, but with a similar kinetic profile to *in vitro*.

In a mouse model of GBM resection, a pilot study was done with GL261-mCh-Luc tumors to evaluate feasibility and dose of perioperative treatment with bolused soluble resiquimod and Ace-Resi (**Supp** Fig. 2A). Two weeks after implant, tumors were debulked and mice were treated with control scaffolds (Ace-Blank), resiquimod loaded scaffolds (Ace-Resi) at a dose of 10 or 100 μg of resiquimod, or a bolus injection of 100 μg resiquimod (Sol. Resi). 80% and 60% of mice treated with Ace-Resi, at low and high doses respectively, achieved durable tumor suppression as measured by bioluminescence and survival (Fig. 1D, **Supp** Fig. 2B–F). However, all mice treated with control scaffold and bolus resiquimod succumbed to tumor burden. Ultimately, bolus injection of resiquimod offered no survival advantage, while sustained exposure to resiquimod did. Treatment with Ace-Resi was well tolerated, with mice experiencing ~ 5–10% transient weight loss for two days following treatment (**Supp** Fig. 2K-L).

To examine immune memory formation, the long-term survivors from both low and high dose Ace-Resi groups were challenged with GL261-mCh-Luc tumors in the contralateral hemisphere 10 weeks after tumor resection and treatment. All mice demonstrated protection from challenge indicating an immune memory response (Fig. 1E, **Supp** Fig. 2G-J). One week after a second tumor challenge, T cell infiltration in the brain was assessed, and an increase in T cell frequency was observed in long-term survivors previously treated with Ace-Resi (Fig. 1F, **Supp** Fig. 2M). Additionally, when Ace-Resi long-term survivors were combined there was an increase in CD8^+^ to CD4^+^ ratio compared to naïve mice bearing tumors (**Supp** Fig. 2N) indicating an increased cytotoxic T cell response.

These results suggested that controlled release via scaffold was more important than dose of resiquimod in achieving durable anti-tumor response. A second study was conducted with an intermediate dose, Ace-Resi (50 μg) (**Supp** Fig. 3A). Similarly to the prior study, this treatment was well tolerated, and 50% of mice treated with Ace-Resi during tumor resection achieved durable tumor suppression (Fig. 1G–H, **Supp** Fig. 3B-C, F-G). These mice were also protected from tumor challenge (Fig. 1I, **Supp** Fig. 3D-E, H). One week after a second tumor challenge, T cell infiltration in the brain was assessed, and a significant increase in CD4^+^ and CD8^+^ T cell frequency was observed in long-term survivors (**Supp** Fig. 3I). In evaluating the phenotype of the T cells, long-term survivors from the Ace-Resi (50 μg) group displayed decreased frequency of naïve and central memory phenotype coupled with increased frequency of effector memory phenotype (Fig. 1J, **Supp** Fig. 3J). Mice treated with the Ace-Resi scaffolds also had an increase in the percentage of granzyme B (GzmB^+^) CD8^+^ T cells (Fig. 1K). This corroborates the indication of an increased cytotoxic T cell response in long-term survivors.

**Figure 2:**
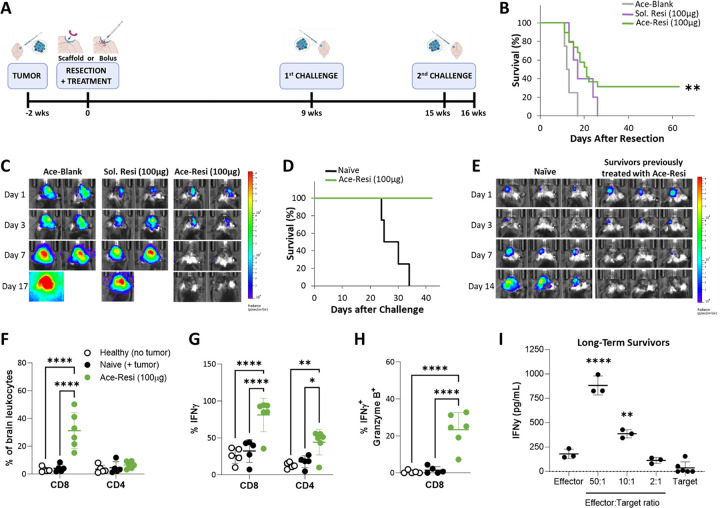
CT2A study **(A)** Timeline of study design. CT2A-Luc tumor implanted and resected 2 weeks later (day 0). Ace-Resi or bolus resiquimod treatment applied during resection. Surviving mice challenged at 9 weeks and then again at 15 weeks. Mice euthanized at 16 weeks for brain lymphocyte analysis. **(B)** Survival of mice after tumor resection and treatment with Ace-Blank, soluble resiquimod (100 μg), or Ace-Resi (100 μg). **p<0.005 by Log-Rank test with respect to Ace-Blank. **(C)** Representative bioluminescent images of mice after tumor resection. **(D)** Survival of mice after first tumor challenge. **(E)** Representative bioluminescent images of mice following first tumor challenge. **(F)** Frequency CD8^+^ or CD4^+^ T cells in brain after second tumor challenge. **(G)** Percent of T cells that are positive for IFNγ or **(H)** CD8^+^ T cells that are positive for both IFNγ and granzyme B. *p<0.05, **p<0.01, ****p<0.0001 by one-way ANOVA and Dunnett’s multiple comparisons test. **(I)** IFNγ in the supernatant of effector cells (isolated from long-term survivors) co-culture with CT2A-Par cells (‘target cells’) for 24 hours. **p<0.01, ****p<0.0001 by one-way ANOVA and Tukey’s multiple comparisons test with respect to all other groups.

For a more comprehensive evaluation, we repeated a therapeutic study in mice bearing CT2A-Luc tumors, a model shown to be more immunologically ‘cold’ than GL261 (Fig. 2A).(32) Like the GL261 model, all mice treated with the control scaffold or bolus resiquimod succumbed to tumor burden. In contrast, perioperative treatment with Ace-Resi not only extended median survival (21.5 days compared to 12.5 days for Ace-Blank control), but also resulted in complete tumor eradication in 31.6% of mice as measured by bioluminescence and survival (Fig. 2B–C, **Supp** Fig. 4A-E). Long-term survivors challenged with CT2A-Luc tumors in the contralateral hemisphere demonstrated complete protection from tumor challenge (Fig. 2D–E, **Supp** Fig. 4F). Brain-infiltrating lymphocytes were evaluated by flow cytometry one week after a second tumor challenge. Mice treated with the Ace-Resi group demonstrated increased frequencies and counts of CD8^+^ T cells (Fig. 2F, **Supp** Fig. 4G), interferon gamma (IFNγ+) CD8^+^ T cells (Fig. 2G, **Supp** Fig. 4H), and polyfunctional IFNγ^+^ GzmB^+^ CD8^+^ T cells (Fig. 2H, **Supp** Fig. 4I) compared to naïve tumor-bearing mice and healthy (no tumor) controls. These results indicated that the implantation of Ace-Resi into the tumor resection cavity induced a robust cytotoxic CD8^+^ T cell response that correlated strongly with protection against tumor rechallenge.

Ace-Resi efficacy was repeated in a CT2A-Par mouse model, a tumor line without any reporter proteins, and displayed comparable survival benefit (**Supp** Fig. 4J-K). Importantly, effector cells isolated from these long-term survivors and co-cultured with CT2A-Par cells released IFNγ in a dose dependent manner (Fig. 2I) whereas effector cells isolated from naïve (non-tumor bearing) mice did not (**Supp** Fig. 4L). This indicates that Ace-Resi induces cytolytic activity in an antigen-specific manner in the absence of reporter protein, luciferin.

### Ace-Resi rapidly remodels the local immune microenvironment and induces systemic T cell trafficking.

**Figure 3:**
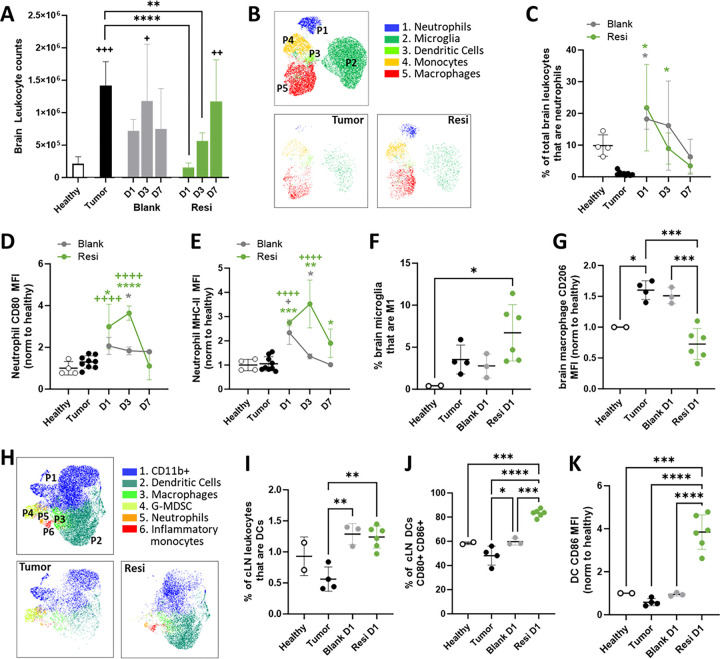
Ace-Resi remodels the local innate immune environment. **(A)** Brain leukocyte counts (*Healthy:* no tumor, no resection; *Tumor:* tumor, no resection; *Blank:* tumor resected, Ace-Blank scaffold; *Resi:* tumor resected, Ace-Resi (100μg) scaffold). **(B)** UMAP of concatenated plot comparing immune populations in the brain of Tumor and Resi mice 3 days after treatment. Immune populations defined based on expression analysis of CD45, CD11b, CD11c, Ly6C, Ly6G, MHCII, CD80, and CD86 as detailed in Supp Fig 13). **(C)** Frequency of neutrophils in the brain. **(D)** Mean fluorescence intensity (MFI) of CD80 and **(E)**MHCII of neutrophils in the brain. **(F)** Percentage of M1 microglia (CD80+ and CD86+) in the brain. **(G)** Normalized MFI of CD206 on macrophages in brain. **(H)** UMAP plot comparing immune populations in the cLN of Tumor and Resi mice 1 day after treatment. Immune populations defined based on expression analysis of CD45, CD11b, CD11c, Ly6C, Ly6G, MHCII, CD80, and CD86 as detailed in Supp Fig 13). **(I)** Percent of cLN leukocytes that are DCs. **(J)** Percent of cLN DCs that are positive for both CD80 and CD86. **(K)** Normalized MFI of CD86 in cLN DC. *p<0.05, **p<0.005, ***p<0.0005, ****p<0.0001 by one-way ANOVA with multiple comparisons Dunnett’s post-hoc test.

We next sought to understand the early immunological events after Ace-Resi treatment. On days 1, 3, and 7 after surgical resection of CT2A-Luc tumors and scaffold treatment, mice were euthanized, and immune populations were analyzed using flow cytometry (**Supp** Fig. 5A). As seen in Fig. 3A and **Supp** Fig. 5B, 24 hours after Ace-Resi treatment, the number of leukocytes in the brain exhibited a precipitous drop and a change in composition, relative to the unresected tumor-bearing control (‘tumor’). While resection and Ace-Blank scaffold also reduced the number of leukocytes, the effect was not statistically significant.

Utilizing hierarchical consensus clustering of flow cytometry data by FlowSOM(47) we identified five distinct populations of immune cells in the brain as shown by UMAP (Fig. 3B, **Supp** Fig. 6A). The most noticeable difference between Ace-Resi and tumor-bearing controls was an increase in the neutrophil population (**Supp** Fig. 6B). This trend was observed for both Ace-Blank and Ace-Resi groups and was most visible three days after resection. Manual gating confirmed a significant increase in neutrophil frequencies in the leukocyte population of Ace-Blank and Ace-Resi treated mice (Fig. 3C). However, while resection and Ace-Blank were sufficient to recruit neutrophils to the brain, Ace-Resi was necessary for sustained upregulation of MHC-II and co-stimulatory molecules CD80 and CD86 (Fig. 3D–E, **Supp** Fig. 5D).(48, 49) Consistent with an acute inflammatory response at the resection site, Ace-Resi treatment significantly increased cytotoxic “M1” microglia (CD80^+^ CD86^+^ microglia, Fig. 3F, **Supp** Fig. 5E) and reduced suppressive macrophages (CD206^+^ macrophages, Fig. 3G, **Supp** Fig. 5F) in the brain creating conditions that promote recruitment and infiltration of peripheral immune cells.(50)

Evaluating the cervical lymph nodes (cLN) one day after resection, we identified six distinct populations of immune cells with hierarchical consensus clustering (Fig. 3H, **Supp Fig. 7A**). This demonstrated a significant increase in the frequency of inflammatory monocytes as well as decreases in neutrophils and granulocytic myeloid derived suppressor cell (G-MDSC) frequencies for Ace-Resi treated mice (**Supp Fig. 7B**). Additionally, both Ace-Resi and Ace-Blank mice had increased frequencies of dendritic cells (DCs) **(**Fig. 3I). However, the DCs trafficking to the cLNs of Ace-Resi treated mice had a distinct activation profile **(Supp Fig. 7A,C)** marked by high expression of co-stimulatory CD80, CD86, and MHCII **(**Fig. 3K, **Supp** Fig. 5G-I**)**. This high expression of CD80 and CD86 was also reflected in the increased frequency of double-positive CD80^+^ and CD86^+^ expression (Fig. 3J).

**Figure 4:**
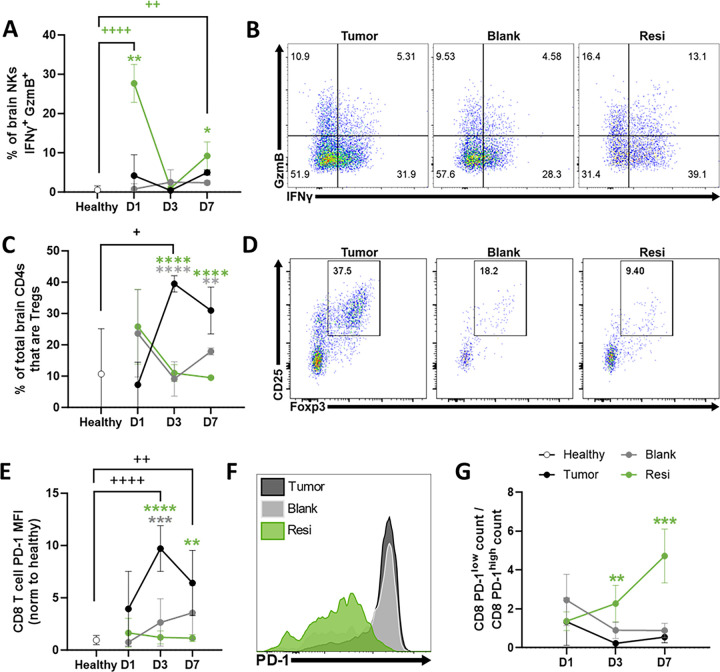
Ace-Resi promotes NK activation, depletes T-regs, and reduces CD8^+^ T cell PD-1 expression. **(A**) Percent of natural killer (NK) cells in the brain that are positive for both IFNγ and granzyme B (GzmB) over time. **(B)** Representative day 7 dot plots demonstrating IFNγ and GzmB expression in NK cells from *Tumor:* tumor-bearing, no resection; *Blank:* tumor resected and Ace-Blank scaffold implanted; *Resi:* tumor resected and Ace-Resi (100μg) scaffold implanted. **(C)** Percent of CD4^+^ T cells that are positive for T-reg markers (Foxp3^+^ CD25^+^) in the brain over time. **(D)** Representative day 7 dot plots of brain-infiltrating Tregs. **(E)** Healthy control normalized mean fluorescent intensity (MFI) and **(F)** day 7 representative histograms of PD-1 staining on brain-infiltrating CD8^+^ T cells. **(G)** Ratio of brain-infiltrating CD8^+^ PD-1^low^ T cell counts to CD8^+^ PD-1^high^ counts. Healthy not shown due to the near absence of PD-1^high^ events. *p<0.05, **p<0.005, ***p<0.0005, ****p<0.0001 by one-way ANOVA with multiple comparisons against the healthy group (+) or tumor group (*) with Dunnett’s post-hoc test.

Examining the early lymphocyte responses to Ace-Resi implantation in the brain, the frequency of highly activated natural killer (NK) cells positive for IFNγ and GzmB was significantly enhanced on both days 1 and 7 in mice (Fig. 4A–B). By day 7, these mice also exhibited high counts of brain-infiltrating double positive IFNγ^+^ GzmB^+^ NK cells (**Supp Fig. 8A**), despite the large drop in brain leukocyte counts immediately following scaffold implantation (Fig. 3A). Interestingly, the TME of untreated tumor-bearing mice also exhibited large numbers of activated NK cells, potentially indicating an active anti-tumor immune response. However, these mice also have high frequencies and counts of regulatory T cell (Treg) populations (Fig. 4C–D, **Supp Fig. 8B**). Consistent with a beneficial role for resection, both Ace-Blank and Ace-Resi treated mice exhibited a significant reduction of Tregs in the brain in contrast to unresected tumor mice (Fig. 4C–D, **Supp Fig. 8B**). Tregs can potently suppress conventional effector T cell responses, and their induction and recruitment to the TME is a major mechanism by which tumors escape immune destruction.(51)

Finally, we examined the phenotype and function of CD8^+^ T cells in the brain. Surprisingly, no significant differences were observed in the frequency of IFNγ^+^ or IFNγ^+^/GzmB^+^ CD8^+^ T cells in the brains of any of the groups that received tumors (**Supp Fig. 8C-E**), and CD8^+^ T cell counts were highest in untreated tumor-bearing mice (**Supp Fig. 8C**). However, despite this active anti-tumor immune response in untreated tumor-bearing mice, tumor growth was unrestrained (Fig. 2C, **Supp** Fig. 4A-B). One mechanism that can restrict CD8^+^ T cell activity is exhaustion; chronic high-level T cell receptor signaling in the TME can cause CD8^+^ T cells to become hyporesponsive to further stimulation, limiting their ability to mediate tumor clearance. This exhausted state is correlated with upregulation of multiple cell surface inhibitory receptors, including PD-1, LAG3, and CTLA4.(52) CD8^+^ T cells from tumor-bearing mice exhibited high surface expression of PD-1, whereas CD8^+^ T cells from Ace-Blank and Ace-Resi mice did not (Fig. 4E–F). Furthermore, when comparing the ratio of PD-1^low^ and PD-1^high^ CD8^+^ T cell counts, mice treated with Ace-Resi exhibited significant skewing towards PD-1^low^ cells over time whereas Ace-Blank alone did not (Fig. 4G).

**Figure 5:**
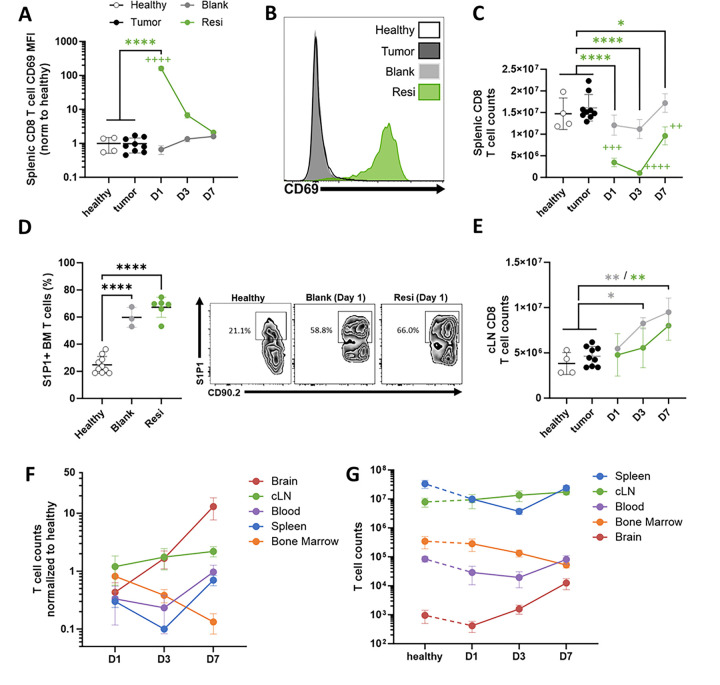
Ace-Resi induces systemic signaling and T cell re-localization. **(A)** Healthy control normalized MFI and **(B)** day 1 representative histograms of CD69 staining on splenic CD8^+^ T cells. **(C)** CD8^+^ T cell counts in the spleen. **(D)** Percentage of S1P1+ T cells from bone marrow (BM) 1 day after resection and representative histogram. **(E)** CD8^+^ T cell counts in the cLN over time. **(F)** T cell counts from mice treated with Ace-Resi in specific organs normalized to ‘healthy’ controls. **(G)** T cell counts from ‘healthy’ controls and mice treated with Ace-Resi in specific organs. Counts from spleen, cLN, and brain are total counts. Counts from blood are per μL. Counts from the bone marrow are total counts in one femur. *p<0.05, **p<0.005, ***p<0.0005, ****p<0.0001 by one-way ANOVA with multiple comparisons with respect to healthy or tumor group (*) or between Ace-Resi and Ace-Blank groups (+) with Tukey’s post-hoc test.

In addition to local brain immune-modulation, we observed rapid systemic effects of Ace-Resi treatment. One day following resection and Ace-Resi implantation, we noted robust CD69 upregulation on T cells in the spleen, bone marrow, and cLN (Fig. 5A–B, **Supp Fig. 9A-B**). In addition to being a marker of T cell activation,(53) CD69 is rapidly upregulated by T cells in response to IFN-I, like IFNβ.(54) Additionally, a marked spike in IFN-β and TNFα was observed at 2 hours which returned to baseline by 24 hours (**Supp Fig. 10**). This was followed by an increase in IFNγ which peaked on day 3 before returning to baseline. We observed transient T cell depletion in the spleens of Ace-Resi treated mice (Fig. 5C), which was not seen in Ace-Blank. Acute viral infection causes similar temporary effects, which are associated with IFN-I-dependent re-localization to the lymph nodes and other tissues.(54)

Intracranial tumors have been shown to induce T cell sequestration in the bone marrow by downregulating the sphingosine-1-phosphate receptor 1 (S1P1), activation of which is required for egress from lymphatic tissue.(19) We observed that Ace-Resi led to a steady decrease in bone marrow T cell counts, concurrently with an increase in T cells in the brain and cLN (Fig. 5E–G). Ace-Blank resulted in similar effects in the bone marrow, but to a lesser degree (**Supp Fig. 9B**). Furthermore, those T cells present within the bone marrow exhibited a significant increase in S1P1 (Fig. 5D), suggesting that surgical resection frees up T cells in the bone marrow for recirculation.

## DISCUSSION

Glioblastoma (GBM) is a highly aggressive brain tumor with a nearly 100% recurrence rate despite standard treatments. Surgical resection is the standard of care for GBM and has profound immune effects; however, preclinical evaluations of immunotherapy are rarely studied within this paradigm.(55, 56) We investigated the effect of continuous local stimulation of TLR7/8 perioperatively by formulating resiquimod within an Ace-DEX polymer scaffold. Scaffolds were fabricated by electrospinning, which produces a thin, flexible, fibrous material ideal for implantation in the brain.(36) Applying the tunability of Ace-DEX polymer platform, we are able to achieve linear release kinetics over 7 days. This is a slower and more controlled release than most formulated resiquimod evaluated in the literature for similar applications which typically only release over 6–12 hours.(57–59)

We observed large differences in the pharmacokinetics of Ace-Resi in the brain versus bolus injection of resiquimod. Ace-Resi had average of 10-fold increases in local resiquimod concentration in the brain compared to serum out to 3 days. Reduced systemic delivery of resiquimod is important as many clinical trials with TLR7/8 stimulation have concerns with systemic toxicities.(60–62) Further reduced dosing of resiquimod may still be efficacious, especially considering that we observed similar long-term protection across resiquimod doses (10, 50, and 100 μg).

In this study we find that sustained local release of resiquimod administered at the time of tumor resection can lead to durable tumor suppression that protects mice from a tumor challenge indicating the formation of immune memory response. It appears that the dose of resiquimod applied is not the critical element to mount an anti-tumor response, but rather that the local resiquimod stimulation last longer than a bolus administration (> 1 hour as measured by pharmacokinetics).

In both GL261-mCh-Luc and CT2A-Luc models, bolus injection of soluble resiquimod intracranially did not result in tumor regression or any long-term survivors. This is in contrast to Grauer et al. who reported that soluble intratumoral injection of 20 μg of resiquimod in an orthotopic GL261 model in the absence of resection, improved survival with ~ 30% of mice clearing their tumor.(28) However, these mice were treated much earlier than in the current study, only 5 days after tumor implant compared to our study that treated 14 days after tumor implant. Additionally, the edema/trauma created by tumor debulking has been found to affect drug diffusion.(45, 46) Therefore it is likely the larger tumor burden, coupled with increased fluid flow induced by surgery, decreased the efficacy of bolus resiquimod in our study.

A similar immunotherapeutic strategy has shown efficacy against other non-intracranial tumors. Park et al. found local resiquimod delivery via hydrogel in orthotopic breast tumors or subcutaneous lung tumors after resection led to improved tumor clearance compared to soluble resiquimod injection.(57) Interestingly, this improved effect was found despite the release of resiquimod from the hydrogel occurring over only 12 hours. Upon investigation into the immunological mechanism, Park et al. determined that anti-tumor effect is driven by IFN-I and both innate and adaptive arms of the immune system are required. However, it should be noted that hydrogels may have limited application for GBM treatment as there have been reports of adverse mass effects for patients.(63–66)

Recently, Turco et al. evaluated a systemic nano formulation of resiquimod against orthotopic GL261 tumors without resection. Utilizing three intravenous administrations of resiquimod nanoparticles (a total dose of 600 μg of resiquimod), they found that treatment led to 43% long-term survivors.(67) It is worth noting that this is comparable to the 80%, 50%, and 60% long-term survivors we achieved with a single administration of 60-, 12-, and 6-fold lower resiquimod dose, respectively, suggesting local administration allows for dose-sparing. This bolsters the strength of our results, confirming that resiquimod is a viable treatment for GBM and warrants further investigation.

Consistent with literature, we found that tumor resection has profound immunostimulatory effects(31, 32) in that it increased DC trafficking to the cLN, inflammatory neutrophils in the brain, and S1P1^+^ expression on T cells in the bone marrow on day 1, with increased CD8^+^ T cells trafficking to the cLN and brain on days 3 and 7. Further, resection decreased Treg populations and PD-1 expression on CD8^+^ T cells. However, many of these effects were transient and all mice treated with resection and control scaffolds succumbed to tumor recurrence. In contrast, resection and Ace-Resi treatment was able to match these effects and continue to build upon them with increased activation of DCs in the cLN, increased polarized microglia and decreased suppressive macrophages in the brain one day after resection. Additionally, Ace-Resi led to an influx of activated NK cells, sustained neutrophil activation out to day 3, and increased the ratio of PD-1^low^ to PD-1^high^ CD8^+^ T cells in the brain.

The encouraging results from this study present several opportunities for further exploration and improvement. Our findings with Ace-Resi and GBM could be more broadly applicable and likely would be similarly effective against brain metastasis of other solid tumors where resection is the standard of care. Encouragingly, mice responded well to this therapy, exhibiting minimal weight loss; however, a comprehensive safety analysis will be essential before translation to a clinical setting. Of note, our finding that resiquimod dose is less important than the sustained local release for antitumor effects, suggests that dose reduction could be achieved for improved safety profile while maintaining therapeutic benefit. Lastly, in this study we utilized a biodegradable polymer scaffold to achieve continuous local stimulation in mice; however, clinically this could be more simply achieved with a catheter (Ommaya reservoir) placed during surgery. This approach could bypass the lengthy FDA approval process for novel biomaterials and provide the added safety benefit of easily suspending treatment if necessary.

**Figure 6:**
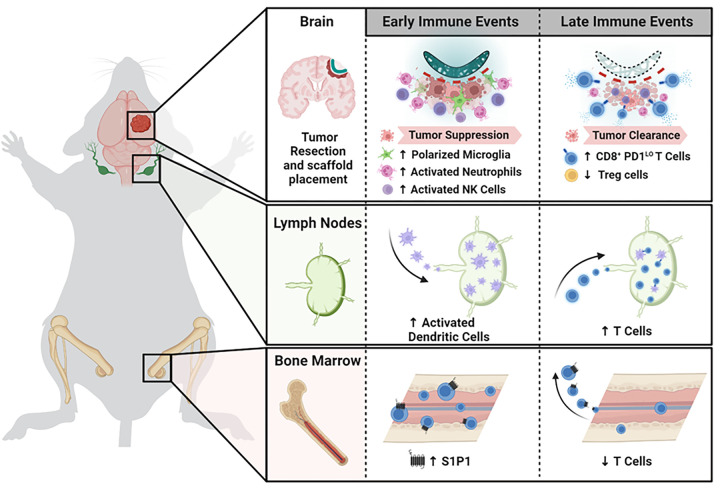
Summary of Ace-Resi effects. Perioperative administration of Ace-Resi scaffold resets the immunosuppressive TME: depleting suppressive regulatory T cells, decreasing M2 macrophages, and polarizing microglia to an inflammatory M1 phenotype. An increase in S1P1 allows T cells to traffic out of the bone marrow and to the cervical lymph node where they can be primed by activated dendritic cells. A wave of inflammatory neutrophils and NK cells traffic to the brain followed by an influx of PD1^low^ CD8^+^ T cells. These events help mount a robust immune response to clear residual tumor and ultimately lead to protection from tumor challenge.

In summary, we formulated TLR7/8 agonist, resiquimod, within a biodegradable nanofibrous Ace-DEX scaffold. We found that scaffolds placed in a sham resection cavity increased and sustained local concentration of resiquimod in the brain. In two murine models of glioma resection and recurrence, GL261 and CT2A, we found that sustained release of resiquimod was necessary to form an anti-tumor immune response and that surviving mice were protected from subsequent tumor challenge. Lastly, we observed that sustained local stimulation of TLR7/8 at the time of tumor resection augments resection-mediated disruption to the immunosuppressive TME and induces systemic T cell trafficking. This allows for the recruitment of inflammatory innate immune cells and freshly primed cytotoxic T cells that can effectively suppress and clear residual tumor while forming a lasting immune memory (Fig. 6).

## MATERIALS AND METHODS

### Experimental Design

The objective of this controlled laboratory experiment was to determine if sustained TLR7/8 agonism (achieved by formulating small molecule, resiquimod, within a biodegradable polymer scaffold) applied at the time of GBM tumor resection would improve survival. Pharmacokinetic studies confirmed that Ace-Resi extended the duration of resiquimod exposure in the murine brain relative to bolus injection. Mice bearing two different orthotopic luminescent GBM tumors (GL261-mCh-Luc and CT2A-Luc) were utilized for evaluation, as well as confirmation in the same cell line without reporter protein (CT2A-Par). Survival was prospectively selected as the primary endpoint, and for luminescent tumors, serial bioluminescence imaging was used to quantify and monitor local tumor recurrence. Formation of an immune memory response towards the tumor was determined by challenging long-term surviving mice with a new inoculate of tumor cells in the contralateral hemisphere utilizing naïve mice as a control. Immune cell infiltrates were evaluated by flow cytometry after a second tumor challenge. Tolerability of Ace-Resi treatment was evaluated by weight loss. For mechanistic studies, flow cytometry and ELISA were used to assess cell populations and soluble cytokines, respectively.

Sample sizes were determined based on results from prior experiments to ensure statistical significance could be analyzed. No animals that received treatment were excluded from the analysis. One control mouse was excluded as it died unrelated to tumor burden. Mice were randomly assigned to treatment groups the day of surgery. When possible, monitoring mice for survival was done in a blinded manner. Mice were evaluated daily and euthanized if they lost more than 20% body weight, or if they exhibited signs of distress or gait abnormalities.

### Chemicals and Reagents

All chemicals were purchased from Sigma-Aldrich (St. Louis, MO) and used as purchased, unless otherwise noted. All disposables were purchased from VWR International (Radnor, PA) unless indicated. High glucose Dulbecco’s Modified Eagle Medium (DMEM) cell culture medium, puromycin dihydrochloride, and fetal bovine serum (FBS) were obtained from Corning (Corning, NY). Penicillin/streptomycin was purchased from Hyclone (Pittsburgh, PA). mCherry-firefly luciferase (mCh-FL) was acquired from Invitrogen (Carlsbad, CA). 2-ethoxypropene was obtained from Matrix Scientific (Columbia, SC). Resiquimod (Resi, > 99%) was acquired from Accel PharmTech (East Brunswick, NJ).

### Cell Culture

GL261 cells were engineered to express mCherry-firefly luciferase (mCh-Luc) using previously developed lentiviral vectors to allow for fluorescent and bioluminescent imaging as in our previous study (‘GL261-mCh-Luc’).(68) Cells were infected for 24 hours and purified by puromycin selection. Murine glioma cell line CT2A expressing firefly luciferase (‘CT2A-Luc’) was purchased from Millipore Sigma (Burlington, MA, Cat # SC195). The parental CT2A cell line (‘CT2A-Par’) was a gift from Professor Thomas Seyfried. Cells were cultured in DMEM medium supplemented with 1% penicillin/streptomycin and 10% fetal bovine serum.

### Scaffold Fabrication

Dextran (molecular weight 450–650 kDa) was chemically modified to generate the hydrophobic biopolymer Ace-DEX as previously described in Kauffman et al.(43) Cyclic acetal coverage (CAC) was determined by NMR.(43) Resiquimod loaded scaffolds were fabricated via electrospinning similar to Graham-Gurysh et al.(37) Briefly, 31% CAC Ace-DEX was dissolved at a concentration of 300 mg/mL in a tri-solvent system consisting of dichloromethane, hexafluoro-2-propanol, and butanol at a ratio of 30%, 30%, and 40% v/v respectively. Resiquimod (10% wt/wt) was added to the polymer solution and extruded from a glass syringe with a 21-guage needle at a flow rate of 1 mL per hour. A voltage bias was applied to the needle and a metal collection plate. The resulting scaffold (termed “Ace-Resi”) was retrieved from the collection plate and stored at −20°C until further use. A scaffold without any resiquimod was fabricated similarly as a control, termed “Ace-Blank”.

### Scaffold Characterization

Electrospun scaffolds were evaluated by scanning electron microscopy to elucidate fiber morphology. A small piece of each scaffold was mounted on aluminum stubs using carbon tape, sputter coated with palladium, and imaged at 2 kV on a Hitachi S-4700 Cold Cathode Field Emission Scanning Electron Microscope (Hitachi High-Technologies, Krefeld, Germany).

To measure resiquimod loading, scaffold samples were weighed, dissolved in DMSO and the absorbance at 260 nm was compared to a standard curve. Resiquimod encapsulation efficiency was defined as the amount of measured resiquimod loaded divided by the theoretical resiquimod loaded. Resiquimod release and scaffold mass loss was determined by adding pre-weighed scaffold samples to a sink of phosphate buffered saline (PBS, pH 7.4) agitated at 37°C. At specific time points, scaffold samples were removed, washed with basic water (water with 0.1% triethylamine), lyophilized, and re-weighed to determine the total scaffold mass loss. Scaffold mass loss was calculated according to **Supp Eq. 1**. Each scaffold sample was then dissolved in DMSO and the absorbance at 260 nm was compared against a standard curve to determine the amount of resiquimod retained in each sample. Resiquimod release was calculated according to **Supp Eq. 2**. Ace-Resi and Ace-Blank scaffolds were tested for endotoxin and measured less than 0.1 EU per mg of scaffold (Pierce Chromogenic Endotoxin Quant Kit).

All experimental protocols were approved by the Animal Care and Use Committees at UNC-CH, and care of the mice was in accordance with the standards set forth by the National Institutes of Health Guide for the Care and Use of Laboratory Animals, and the American Veterinary Medical Association. To assess the pharmacokinetics of resiquimod released from Ace-Resi/fast within the mouse brain compared to bolus soluble injection, experimental groups were applied to a resection cavity created in the right frontal lobe in a non-tumor bearing C57BL/6 mouse. At 1hr, 6hr, 1, 3, and 7 days, mice were euthanized, and brain and plasma collected. Any remaining scaffold was removed from the brain. The remaining scaffold was lyophilized, dissolved in DMSO, and evaluated by fluorescence (excitation 260nm, emission 370nm) against a standard curve to quantitate the percentage of resiquimod retained within the scaffold.

Plasma samples and brain tissue were snap frozen. Brain tissue was homogenized in PBS using 1.4 mm porcelain homogenization beads and diluted in PBS for a 4x dilution. Samples were analyzed with LC-MS/MS to measure the concentration of resiquimod. The lower limit of quantification for resiquimod in the brain was 1 ng per gram of tissue and in plasma was 1 ng per mL.

### Glioblastoma mouse model

C57BL/6 mice were purchased from Charles River (stock# 027) or Jackson Labs (stock# 00064) for GL261 and CT-2A models, respectively. For all surgical procedures, mice were anesthetized by vapor isoflurane. Bupivacaine was utilized for local analgesia and meloxicam (5 mg/kg) was administered prior to surgery and once daily for the following 3 days. Mice were evaluated daily and euthanized if they lost more than 20% body weight, or if they exhibited signs of distress or gait abnormalities.

A model of GBM resection and recurrence was used to evaluate drug-loaded scaffolds similar to previously described.(37) Complete timelines for all studies are in the main text or supplement (Fig. 2A, **Supp** Fig. 2A, 3A, 4J, 5A). GBM tumor cells were injected (either GL261-mCh-Luc, CT2A-Luc, or CT2A-Par) at 1 × 10^5^ cells in 2 μL of PBS at a rate of 1 μL per minute into the right frontal lobe of the brain via burr hole approximately 2mm lateral of bregma and 0.5 mm below the dura. For luminescent tumors, growth was monitored by bioluminescent imaging (BLI, Perkin Elmer IVIS Spectrum In Vivo Imaging System). Ten to 14 days after implantation, a cranial window ~ 3mm in diameter was created around the initial burr hole. Tumors were then partially resected, treatment was applied to the surgical cavity, and the skin was closed with Vetbond tissue adhesive (3M, Maplewood, MN). For mice treated with a bolus soluble resiquimod, 100 μg of resiquimod in 5 μL of PBS and DMSO (80%, 20% v/v) was injected into the exposed brain tissue of the resection cavity. For mice treated with scaffolds, the scaffolds were simply placed into the resection cavity. The resiquimod dose implanted via Ace-Resi scaffolds was controlled by changing mass of scaffold implanted knowing the empirical drug loading. With a 9.5% w/w loaded scaffold, a 100 μg dose of resiquimod would require 1.05 mg of scaffold, whereas a 10 μg dose of resiquimod would require 0.11 mg of scaffold. Sample sizes for each study are detailed in **Supp Table 2**. Mice were monitored for tumor recurrence and survival. Serial BLI was used to quantify tumor growth starting the day after tumor resection (Day 1).

For GL261-mCh-Luc and CT2A-Luc studies, to assess for a memory immune response, survivors were challenged with tumors (2 × 10^5^ GL261-mCh-Luc or CT2A-Luc) in the contralateral hemisphere 9–10 weeks after initial resection and treatment. As a control, naïve mice were also implanted with tumors. Mice were followed for survival and 6–7 weeks later, survivors were re-challenged. One week after the second challenge, mice were euthanized and brain leukocytes were analyzed by flow cytometry.

For the CT2A-Par study, survivors were challenged with 2 × 10^5^ CT2A-Par tumors in the contralateral hemisphere 9 weeks after resection and treatment. One week after tumor challenge, mice were euthanized, and brains and one spleen were collected. Leukocytes were isolated from the pooled brains and CD8^+^ T cells isolated from the spleen via magnetic bead separation (Miltenyi cat # 130-104-075). Additionally, as a control a healthy naïve mouse was also euthanized and CD8^+^ T cells were isolated from the spleen. T cells from both groups were expanded through 48hr culture on CD3 and CD28 antibody coated plates (0.5 μg per well, 4hr incubation at 37°C). For long-term survivors, brain leukocytes (accounting for 5% of the total effector population) and CD8^+^ T cells isolated from spleen (accounting for 95% of the effector cells) were cocultured with CT2A-Par cells at various ratios. For naïve mice, CD8^+^ T cells from the spleen (accounting for 100% of the effector population) were cocultured with CT2A-Par cells at various ratios. Media was collected at 24 hours from the coculture and evaluated by cytokine ELISA for IFNγ (Biolegend Cat # 430801).

### Evaluation of Early Immunological Effects

Mice were implanted with CT2A-Luc tumors in the right frontal lobe as described above. Two weeks later, tumors were resected, and scaffolds implanted with the following groups: Ace-Blank, Ace-Resi 100 μg. As controls, a subset of mice with and without tumors did not undergo resection, ‘tumor’ and ‘healthy’ respectively. Sample sizes for these groups are detailed in **Supp Table 2**. At days 1, 3, and 7 following resection mice were euthanized. Brain, cervical lymph nodes, spleen, bone marrow, and blood were collected for leukocyte analysis. Cytokines were measured through ELISA according to manufacturer guidelines: IFN-β (R&D Systems Catalog # DY8234-05), TNF-α (Biolegend Cat # 430901), IFN-γ (Biolegend Cat # 430801).

Cervical lymph nodes, spleen, and bone marrow (extruded from a single femur and tibia) were harvested. Single cell suspensions were generated by grinding through a 70-μm cell strainer. Red blood cells were lysed from spleen and bone marrow samples with ACK lysis buffer. All cell suspensions were washed with PBS + 2% fetal bovine serum (FACs buffer), then passed through another 70 μm strainer. Blood was collected into both K3EDTA coated and serum separator tubes (Greiner). Serum was stored at −80°C for later use. Fifty μL of blood from K3EDTA tubes was treated with ACK lysis buffer to remove red blood cells. The cell suspension was then washed with FACs buffer.

For S1P1 staining, bone marrow (extruded from a single femur) was harvested into S1P1 buffer (500 mL PBS, 5 mL 10% Buffered Neutral Formalin, 2 mL 0.5 M EDTA, 2.5 g BSA) to immediately fix cells. Single cell suspensions were generated by grinding through a 70-μm cell strainer in S1P1 buffer. Red blood cells were lysed from spleen and bone marrow samples with ACK lysis buffer. Cell suspensions were washed with S1P1 buffer and passed through another 70 μm strainer. Cells were washed twice in FACS buffer, stained for 30 minutes on ice, then washed twice and resuspended in FACS buffer.

Whole brains were removed and dissociated via homogenization in Hank’s buffered salt solution (HBSS). For lymphocyte evaluation 7 days after tumor challenges in GL261-mCh-Luc and CT2A-Luc models, brain homogenate was resolved with a 70%, 30% Percoll gradient, collecting single cells at the interface. For leukocyte analysis of the early immunological effects on days 1, 3, and 7 following treatment, brain homogenate was suspended in 30% Percoll gradient and the cell pellet was collected.

Following generation of single-cell suspensions, cells were divided based on downstream application (either surface staining or intracellular cytokine staining (ICCS)). For the identification and exclusion of dead cells in all flow analysis, samples were stained with eBioscience Fixable Viability Dye (ThermoFisher) diluted 1:1000 in FACS buffer for 30 minutes on ice, then washed twice with FACS buffer. All samples were acquired using either an Attune NxT flow cytometer (ThermoFisher) or an LSRFortessa flow cytometer (Becton Dickinson). Flow data analysis was performed using FlowJo (Becton Dickinson). All samples were subject to sequential debris, doublet, and dead cell removal prior to analysis. A complete list of antibody clones, fluorochrome conjugates, vendors, and dilutions is included in **Supp Table 3.**

For ‘myeloid’, ‘systemic lymphocyte’, and ‘microglia and macrophage’ panels (involving only surface stains), cells were washed twice in FACS buffer, then stained for 30 minutes on ice in FACS buffer, resuspended in 1% formaldehyde in PBS for 30 minutes on ice, then washed twice and resuspended in FACS buffer.

For ICCS, cells were treated with brefeldin A and monensin (5 μg/mL and 2 μM, Biolegend) with and without phorbol 12-myristate 13-acetate and ionomycin (50 ng/mL and 500 ng/mL, Sigma) for 4 hours at 37°C. Cells were then fixed and permeabilized for 30 minutes on ice using the eBioscience^™^ Foxp3 / Transcription Factor Staining Buffer Set (Thermo Fisher), washed twice with permeabilization buffer, then stained with a mixture of fluorochrome-conjugated antibodies diluted in permeabilization buffer for 30 minutes at room temperature, washed twice with permeabilization buffer and resuspended in FACS buffer.

Myeloid panel: Cells expressing high levels of CD45 were selected for downstream analysis Leukocytes (CD45^high^), Lymphocytes (CD45^high^ TCR-β/CD19^+^), DCs (CD45^high^ TCR-β/CD19^−^ CD11c^+^ MHC-II^+^), Monocytes (CD45^high^ TCR-β/CD19^−^ CD11b^high^ Ly6C^+^), Macrophages (CD45^high^ TCR-β/CD19^−^ CD11b^high^ Ly6C^−^), Neutrophils (CD45^high^ TCR-β/CD19^−^ CD11b^high^ Ly6G^+^), CD11b^int^ (CD45^high^ TCR-β/CD19^−^ CD11b^int^), and Lin^−^ (Negative for other lineage-defining markers). Cell types were assessed for MFI of CD80, CD86, and MHC-II. The gating strategy for this panel is in **Supp Fig. 11**.

Microglia and macrophage panel: Cells expressing high levels of CD45 were selected for downstream analysis and microglia were identified through expression of an intermediate CD45 signal (CD45^int^). Lymphocytes (CD19^+^ CD3^+^ TCRß^+^) were removed through a dump channel (PE/Dazzle594). Non-lymphocytes were subject to CD11b stratification, and the high-expressing population selected. This population (CD45^high^ CD19^−^ CD3^−^ TCRß^−^ CD11b^high^) designated macrophages. Macrophages and microglia were assessed for frequency and MFI of CD80, CD86, and CD206. The gating strategy for this panel is in **Supp Fig. 12**.

Systemic lymphocyte panel: B cells (CD19^+^) and NK cells (NK1.1^+^) were removed via dump channel and CD4^+^ T cells (CD90.2^+^ CD4^+^) CD8^+^ T cells (CD90.2^+^ CD8^+^) were identified. T cell central memory (CD44^+^ CD62L^+^), effector memory (CD44^+^ CD62L^−^), and naïve (CD44^−^ CD62L^+/−^) subpopulations were then stratified. CD69^+^ and CD44 MFI were analyzed for both CD4 and CD8^+^ T cells. Total T-cell population S1P1^+^ differences were assessed by selecting for CD90.2^+^ CD19^−^ cells (combined CD4s and CD8s).

Brain lymphocyte panel: NK cells (CD90.2^+^ NK1.1^+^), CD4^+^ T cells (CD90.2^+^ CD4^+^), and CD8^+^ T cells (CD90.2^+^ CD8^+^) populations were identified. T cell central memory (CD44^+^ CD62L^+^), effector memory (CD44^+^ CD62L^−^), and naïve (CD44^−^ CD62L^+/−^) subpopulations were then stratified. CD25^+^, IFN-γ^+^, GzmB^+^, PD1^+^ frequency and MFI were analyzed for NK cells and CD8^+^ T cells. IFN-γ^+^, FoxP3^+^, IL-10^+^ frequency and MFI were analyzed for CD4^+^ T cells.

### FlowSOM UMAP Analysis

Following the gating strategy in **Supplemental Fig. 13B-C**, the flow cytometry data for the myeloid cell panel were down-sampled on a CD45 + gate to ensure each animal was represented equally. Files were concatenated to generate UMAP figures clustered by FlowSOM for the brain and cervical lymph node. FlowSOM clusters were defined via a heatmap in **Supplemental Fig. 13D-E.**

### Statistical Analysis

Statistical analysis was performed using GraphPad Prism (La Jolla, CA). Mouse survival curves were evaluated by Log-Rank (Mantel-Cox) test. Statistical significance for flow cytometry data was determined by one- or two-way ANOVA, with multiple comparisons using Tukey’s or Dunnett’s post-hoc test. When no difference was seen temporally across days 1, 3, and 7, non-resected ‘tumor’ mice results were grouped together to increase power.
